# Crystal structure of *N*-[(methyl­sulfan­yl)carbon­yl]urea

**DOI:** 10.1107/S2056989016002322

**Published:** 2016-02-13

**Authors:** Mouhamadou Birame Diop, Libasse Diop, Allen G. Oliver

**Affiliations:** a Laboratoire de Chimie Minérale et Analytique, Département de Chimie, Faculté des Sciences et Techniques, Université Cheikh Anta Diop, Dakar, Senegal; b Department of Chemistry and Biochemistry, University of Notre Dame, Notre Dame, IN 46557-5670, USA

**Keywords:** crystal structure, hydrogen bonds, one-dimensional structure

## Abstract

The almost planar (r.m.s. deviation = 0.055 Å) title compound, (MeS)C(O)NHC(O)NH_2_, was formed during an attempted crystallization of dimethyl cyano­carbonimidodi­thio­ate with CrO_2_Cl_2_; an unexpected redox reaction converted the cyano­carbonimido moiety to a urea group and removed one methyl­thiol group. In the crystal, hydrogen-bonding inter­actions from the amide and amido N—H groups to carbonyl O atoms of neighbouring mol­ecules result in [010] ribbon-like chains.

## Abstract 2

In the crystal of (MeS)C(O)NHC(O)NH_2_, the mol­ecules are connected *via* N—H⋯O hydrogen bonds, forming ribbon-like chains.

## Chemical context   

We have recently reported that dimethyl cyano­carbon­imido­di­thio­ate (MeS)_2_C=N—C N is an N-donor ligand, coord­in­ating to metal centres (Diop *et al.*, 2016 ▸). In an attempt to broaden data on the coordination ability of this ligand, we have initiated here a study of the inter­actions between dimethyl cyano­carbonimidodi­thio­ate and CrO_2_Cl_2_ which yielded the title compound whose X-ray study is reported in this work. Surprisingly, the dimethyl cyano­carbonimidodi­thio­ate has undergone redox reactivity at both the cyanide (N1/C1) and the imido (N2/C2) functionalities. The carbon atoms associated with these groups have been oxidized to an amide and both nitro­gen atoms now sport hydrogen atoms. One methyl­thiol group has been removed during this reaction. Presumably adventitious water is the source of the oxygen and hydrogen. This was unexpected reactivity. It is not known if or how the CrO_2_Cl_2_ plays a role in this reaction.
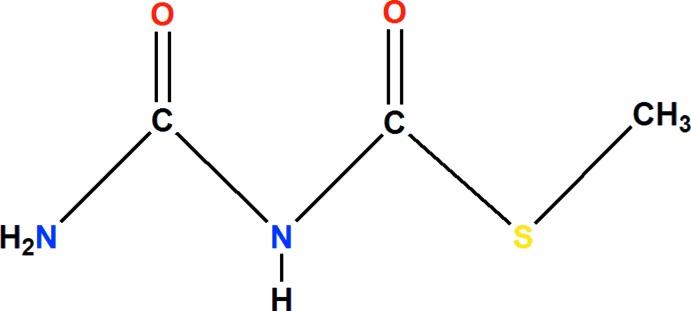


## Structural commentary   

**Figure 1:**
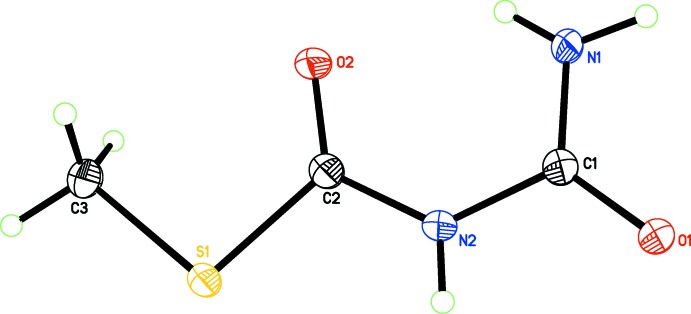
The mol­ecular structure of the title compound. Displacement ellipsoids are depicted at the 50% probability level and H atoms as spheres of an arbitrary radius.

The starting dimethyl cyano­carbonimidodi­thio­ate (MeS)_2_C=N—C N has undergone oxidation yielding the title compound (MeS)C(O)NHC(O)NH_2_ (Fig. 1 ▸). Bond distances and angles within the mol­ecule are in the expected range (Sow *et al.*, 2014 ▸; Jalový *et al.*, 2011 ▸). Although the C1—N1 [1.3159 (19) Å] bond appears shorter than the C2—N2 [1.3623 (18) Å] and C1—N2 [1.3977 (18) Å] bonds, all three are within expected ranges for urea N—C bond distances (*MOGUL* analysis; Bruno *et al.*, 2004 ▸) because of the different substituents on the carbon atoms. The C2—S1—C3 bond angle is 99.22 (7)°. The torsion angles are close to zero or 180°, which is consistent with a nearly planar mol­ecule (r.m.s. deviation for the non-hydrogen atoms = 0.055 Å). An intra­molecular N1—H1*NB*⋯O2 hydrogen bond generates an *S*(6) ring (see Table 1 ▸).

## Supra­molecular features   

**Figure 2:**
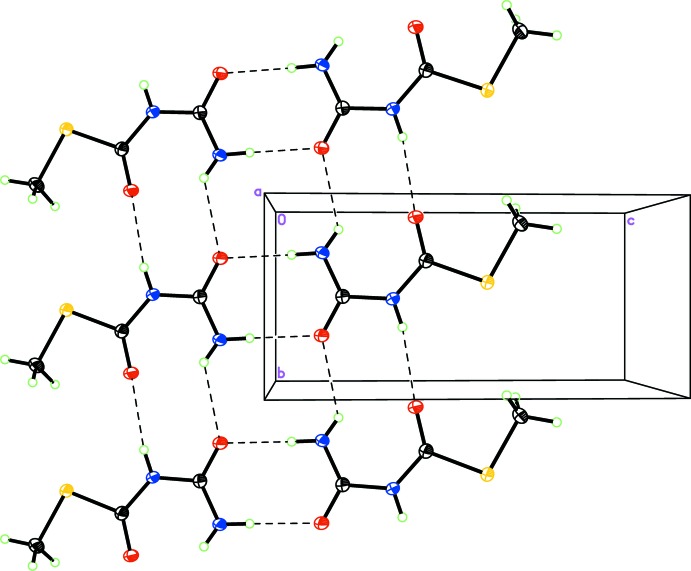
Packing diagram of the title compound showing one-dimensional hydrogen-bonded chains (dashed lines) viewed along the *a* axis.

In the crystal, the compound forms a hydrogen-bonded dimer with a mol­ecule related through the inversion center at (½, ½, 0) [N1⋯O1^ii^; symmetry code: (ii) −*x* + 1, −*y* + 1, −*z*). This ‘head-to-head’ arrangement forces the non-inter­acting thio­methyl groups to be on the exterior of the chain. These hydrogen-bonded dimers propagate into a one-dimensional chain parallel to the *b* axis (Fig. 2 ▸) through hydrogen bonds from N1⋯O1^i^ and N2⋯O2^iii^ [symmetry codes: (i) *x*, *y* − 1, *z*; (iii) *x*, *y* + 1, *z*]. The ribbons are oriented approximately parallel to the [30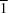] plane. The compactness and the stability of the structure are consolidated through van der Waals forces and weak C—H⋯O and C—H⋯S hydrogen bonds(Table 1 ▸).

## Database survey   

To the best of our knowledge there are no reported structures that contain the *N*-[(methylsulfanyl)carbonyl]urea group (CSD Version 5.37 plus one update; Groom &Allen, 2014 ▸).

## Synthesis and crystallization   

All chemicals are purchased from Aldrich Company (Germany) and used as received. Dimethyl cyano­carbon­imido­di­thio­ate was mixed in aceto­nitrile with CrO_2_Cl_2_ in a 1:1 ratio: a green solution was obtained. Two colourless crystals – one of which being this studied compound – suitable for a single-crystal X-ray diffraction study were obtained after a slow solvent evaporation at room temperature (303 K).

## Refinement   

Crystal data, data collection and structure refinement details are summarized in Table 2 ▸. Urea hydrogen atoms were located from a difference Fourier map and refined freely. Methyl hydrogen atoms were included in geometrically calculated positions and allowed to rotate to minimize their contribution to electron density with C—H = 0.98 Å and *U*_iso_(H) = 1.5*U*_eq_(C3).

## Supplementary Material

Crystal structure: contains datablock(s) I. DOI: 10.1107/S2056989016002322/hb7563sup1.cif

Structure factors: contains datablock(s) I. DOI: 10.1107/S2056989016002322/hb7563Isup2.hkl

Supporting information file. DOI: 10.1107/S2056989016002322/hb7563Isup3.cml

CCDC reference: 1452062

Additional supporting information:  crystallographic information; 3D view; checkCIF report

## Figures and Tables

**Table 1:** Hydrogen-bond geometry (Å, °)

*D*—H⋯*A*	*D*—H	H⋯*A*	*D*⋯*A*	*D*—H⋯*A*
N1—H1*NB*⋯O1^i^	0.77 (2)	2.27 (2)	2.8518 (16)	132.3 (19)
N1—H1*NB*⋯O2	0.77 (2)	2.15 (2)	2.7397 (17)	134 (2)
N1—H1*NA*⋯O1^ii^	0.87 (2)	2.05 (2)	2.9221 (16)	178 (2)
N2—H2*NA*⋯O2^iii^	0.805 (19)	2.18 (2)	2.9709 (15)	168.9 (16)
C3—H3*A*⋯O2^iv^	0.98	2.54	3.494 (2)	166
C3—H3*B*⋯S1^v^	0.98	2.85	3.7064 (15)	147

Symmetry codes: (i) 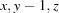; (ii) 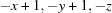; (iii) 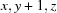; (iv) 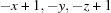; (v) 
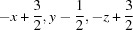
.

**Table 2:** Experimental details

Crystal data	
Chemical formula	C_3_H_6_N_2_O_2_S
*M*_r_	134.16
Crystal system, space group	Monoclinic, *P*2_1_/*n*
Temperature (K)	120
*a*, *b*, *c* (Å)	9.9388 (13), 5.0999 (6), 10.6755 (14)
β (°)	94.136 (4)
*V* (Å^3^)	539.70 (12)
*Z*	4
Radiation type	Mo *K*α
μ (mm^−1^)	0.50
Crystal size (mm)	0.24 × 0.19 × 0.14

Data collection	
Diffractometer	Bruker Kappa X8–APEXII
Absorption correction	Multi-scan (*SADABS*; Krause *et al.*, 2015 ▸)
*T*_min_, *T*_max_	0.679, 0.734
No. of measured, independent and observed [*I* > 2σ(*I*)] reflections	8437, 1344, 1220
*R*_int_	0.024
(sin θ/λ)_max_ (Å^−1^)	0.669

Refinement	
*R*[*F* ^2^ > 2σ(*F* ^2^)], *wR*(*F* ^2^), *S*	0.031, 0.084, 1.09
No. of reflections	1344
No. of parameters	86
H-atom treatment	H atoms treated by a mixture of independent and constrained refinement
Δρ_max_, Δρ_min_ (e Å^−3^)	0.38, −0.23

Computer programs: *APEX3* (Bruker, 2015 ▸), *SAINT* (Bruker, 2015 ▸), *SHELXT2014* (Sheldrick, 2015*a* ▸), *SHELXL2014* (Sheldrick, 2015*b* ▸), *XP* in *SHELXTL* (Sheldrick, 2008 ▸) and *publCIF* (Westrip, 2010 ▸).

## supplementary crystallographic information

### Crystal data

**Table:**

C_3_H_6_N_2_O_2_S	*F*(000) = 280
*M**_r_* = 134.16	*D*_x_ = 1.651 Mg m^−^^3^
Monoclinic, *P*2_1_/*n*	Mo *K*α radiation, λ = 0.71073 Å
*a* = 9.9388 (13) Å	Cell parameters from 3996 reflections
*b* = 5.0999 (6) Å	θ = 2.7–28.3°
*c* = 10.6755 (14) Å	µ = 0.50 mm^−^^1^
β = 94.136 (4)°	*T* = 120 K
*V* = 539.70 (12) Å^3^	Tablet, colorless
*Z* = 4	0.24 × 0.19 × 0.14 mm

### Data collection

**Table:**

Bruker Kappa X8-APEXII diffractometer	1344 independent reflections
Radiation source: fine-focus sealed tube	1220 reflections with *I* > 2σ(*I*)
Graphite monochromator	*R*_int_ = 0.024
Detector resolution: 8.33 pixels mm^-1^	θ_max_ = 28.4°, θ_min_ = 2.7°
combination of ω and φ–scans	*h* = −13→13
Absorption correction: multi-scan (*SADABS*; Krause *et al.*, 2015)	*k* = −6→6
*T*_min_ = 0.679, *T*_max_ = 0.734	*l* = −8→14
8437 measured reflections	

### Refinement

**Table:**

Refinement on *F*^2^	Primary atom site location: real-space vector search
Least-squares matrix: full	Secondary atom site location: difference Fourier map
*R*[*F*^2^ > 2σ(*F*^2^)] = 0.031	Hydrogen site location: mixed
*wR*(*F*^2^) = 0.084	H atoms treated by a mixture of independent and constrained refinement
*S* = 1.09	*w* = 1/[σ^2^(*F*_o_^2^) + (0.0502*P*)^2^ + 0.2127*P*] where *P* = (*F*_o_^2^ + 2*F*_c_^2^)/3
1344 reflections	(Δ/σ)_max_ = 0.001
86 parameters	Δρ_max_ = 0.38 e Å^−^^3^
0 restraints	Δρ_min_ = −0.23 e Å^−^^3^

### Special details

**Table:**

Geometry. All e.s.d.'s (except the e.s.d. in the dihedral angle between two l.s. planes) are estimated using the full covariance matrix. The cell e.s.d.'s are taken into account individually in the estimation of e.s.d.'s in distances, angles and torsion angles; correlations between e.s.d.'s in cell parameters are only used when they are defined by crystal symmetry. An approximate (isotropic) treatment of cell e.s.d.'s is used for estimating e.s.d.'s involving l.s. planes.

### Fractional atomic coordinates and isotropic or equivalent isotropic displacement parameters (Å^2^)

**Table:**

	*x*	*y*	*z*	*U*_iso_*/*U*_eq_	
S1	0.69406 (4)	0.42571 (7)	0.54507 (3)	0.01914 (14)	
O1	0.54686 (11)	0.7074 (2)	0.13127 (9)	0.0204 (2)	
O2	0.62689 (10)	0.08127 (18)	0.36859 (10)	0.0183 (2)	
N1	0.55218 (13)	0.2665 (2)	0.13289 (12)	0.0186 (3)	
H1NB	0.5637 (18)	0.144 (5)	0.1744 (19)	0.025 (5)*	
H1NA	0.521 (2)	0.276 (5)	0.055 (2)	0.036 (5)*	
N2	0.61955 (13)	0.5113 (2)	0.31095 (12)	0.0162 (3)	
H2NA	0.6285 (16)	0.660 (4)	0.3351 (16)	0.013 (4)*	
C1	0.57029 (14)	0.4988 (3)	0.18511 (13)	0.0157 (3)	
C2	0.64159 (13)	0.3108 (3)	0.39424 (12)	0.0154 (3)	
C3	0.70883 (16)	0.1190 (3)	0.62609 (14)	0.0229 (3)	
H3A	0.6205	0.0327	0.6228	0.034*	
H3B	0.7408	0.1494	0.7139	0.034*	
H3C	0.7732	0.0067	0.5860	0.034*	

### Atomic displacement parameters (Å^2^)

**Table:**

	*U*^11^	*U*^22^	*U*^33^	*U*^12^	*U*^13^	*U*^23^
S1	0.0303 (2)	0.0137 (2)	0.0128 (2)	−0.00005 (12)	−0.00299 (14)	−0.00060 (12)
O1	0.0322 (6)	0.0120 (5)	0.0162 (5)	0.0004 (4)	−0.0045 (4)	0.0002 (4)
O2	0.0258 (5)	0.0117 (5)	0.0169 (5)	−0.0011 (4)	−0.0023 (4)	−0.0009 (4)
N1	0.0296 (7)	0.0110 (6)	0.0143 (6)	0.0009 (5)	−0.0044 (5)	0.0012 (5)
N2	0.0234 (6)	0.0112 (6)	0.0137 (6)	−0.0014 (4)	−0.0009 (4)	−0.0014 (4)
C1	0.0182 (6)	0.0146 (6)	0.0141 (6)	0.0002 (5)	0.0003 (5)	0.0000 (5)
C2	0.0165 (6)	0.0153 (6)	0.0143 (6)	−0.0001 (5)	0.0000 (5)	−0.0006 (5)
C3	0.0336 (8)	0.0172 (7)	0.0170 (7)	−0.0016 (6)	−0.0037 (6)	0.0041 (5)

### Geometric parameters (Å, º)

**Table:**

S1—C2	1.7569 (14)	C2—N2	1.3623 (18)
S1—C3	1.7885 (15)	C1—N2	1.3977 (18)
C1—O1	1.2239 (18)	N2—H2NA	0.805 (19)
C2—O2	1.2088 (17)	C3—H3A	0.9800
C1—N1	1.3159 (19)	C3—H3B	0.9800
N1—H1NB	0.77 (2)	C3—H3C	0.9800
N1—H1NA	0.87 (2)		
			
C2—S1—C3	99.22 (7)	O2—C2—N2	124.61 (13)
C1—N1—H1NB	118.6 (16)	O2—C2—S1	123.61 (11)
C1—N1—H1NA	112.5 (16)	N2—C2—S1	111.78 (10)
H1NB—N1—H1NA	129 (2)	S1—C3—H3A	109.5
C2—N2—C1	128.44 (12)	S1—C3—H3B	109.5
C2—N2—H2NA	119.3 (12)	H3A—C3—H3B	109.5
C1—N2—H2NA	112.0 (12)	S1—C3—H3C	109.5
O1—C1—N1	124.62 (13)	H3A—C3—H3C	109.5
O1—C1—N2	116.95 (13)	H3B—C3—H3C	109.5
N1—C1—N2	118.43 (13)		
			
C2—N2—C1—O1	173.40 (14)	C1—N2—C2—S1	−176.11 (11)
C2—N2—C1—N1	−6.5 (2)	C3—S1—C2—O2	−1.02 (14)
C1—N2—C2—O2	3.7 (2)	C3—S1—C2—N2	178.83 (11)

### Hydrogen-bond geometry (Å, º)

**Table:**

*D*—H···*A*	*D*—H	H···*A*	*D*···*A*	*D*—H···*A*
N1—H1*NB*···O1^i^	0.77 (2)	2.27 (2)	2.8518 (16)	132.3 (19)
N1—H1*NB*···O2	0.77 (2)	2.15 (2)	2.7397 (17)	134 (2)
N1—H1*NA*···O1^ii^	0.87 (2)	2.05 (2)	2.9221 (16)	178 (2)
N2—H2*NA*···O2^iii^	0.805 (19)	2.18 (2)	2.9709 (15)	168.9 (16)
C3—H3*A*···O2^iv^	0.98	2.54	3.494 (2)	166
C3—H3*B*···S1^v^	0.98	2.85	3.7064 (15)	147

Symmetry codes: (i) *x*, *y*−1, *z*; (ii) −*x*+1, −*y*+1, −*z*; (iii) *x*, *y*+1, *z*; (iv) −*x*+1, −*y*, −*z*+1; (v) −*x*+3/2, *y*−1/2, −*z*+3/2.

## References

[bb1] Bruker (2015). *APEX3* and *SAINT*. Bruker–Nonius AXS Inc., Madison, Wisconsin, USA.

[bb2] Bruno, I. J., Cole, J. C., Kessler, M., Luo, J., Motherwell, W. D. S., Purkis, L. H., Smith, B. R., Taylor, R., Cooper, R. I., Harris, S. E. & Orpen, A. G. (2004). *J. Chem. Inf. Model.* **44**, 2133–2144. 10.1021/ci049780b 15554684

[bb3] Diop, M. B., Diop, L. & Oliver, A. G. (2016). *Acta Cryst.* E**72**, 66–68. 10.1107/S2056989015023439 PMC4704751 26870588

[bb4] Groom, C. R. & Allen, F. H. (2014). *Angew. Chem. Int. Ed.* **53**, 662–671. 10.1002/anie.201306438 24382699

[bb5] Jalový, Z., Matyáš, R., Ottis, J., Růžička, A., Šimůnek, P. & Polášek, M. (2011). *Chem. Cent. J.* **5**, 84–94. 10.1186/1752-153X-5-84 PMC3275501 22152129

[bb6] Krause, L., Herbst-Irmer, R., Sheldrick, G. M. & Stalke, D. (2015). *J. Appl. Cryst.* **48**, 3–10. 10.1107/S1600576714022985 PMC4453166 26089746

[bb7] Sheldrick, G. M. (2008). *Acta Cryst.* A**64**, 112–122. 10.1107/S0108767307043930 18156677

[bb8] Sheldrick, G. M. (2015*a*). *Acta Cryst.* A**71**, 3–8.

[bb9] Sheldrick, G. M. (2015*b*). *Acta Cryst.* C**71**, 3–8.

[bb10] Sow, Y., Diop, L., Fernandes, M. A. & Stoeckli-Evans, H. (2014). *Acta Cryst.* E**70**, m83. 10.1107/S1600536814002025 PMC3998430 24764948

[bb11] Westrip, S. P. (2010). *J. Appl. Cryst.* **43**, 920–925.

